# Creatinine Change on Vasoconstrictors as Mortality Surrogate in Hepatorenal Syndrome: Systematic Review & Meta-Analysis

**DOI:** 10.1371/journal.pone.0135625

**Published:** 2015-08-21

**Authors:** Justin M. Belcher, Steven G. Coca, Chirag R. Parikh

**Affiliations:** 1 Program of Applied Translational Research, Yale University School of Medicine, New Haven, CT, United States of America; 2 Section of Nephrology, Yale University School of Medicine, New Haven, CT, United States of America; 3 Clinical Epidemiology Research Center, VAMC, West Haven, CT, United States of America; Taipei Veterans General Hospital, TAIWAN

## Abstract

**Background and Aims:**

Hepatorenal syndrome is a severe complication of cirrhosis and associates with significant mortality. Vasoconstrictor medications improve renal function in patients with hepatorenal syndrome. However, it is unclear to what extent changes in serum creatinine during treatment may act as a surrogate for changes in mortality. We have performed a meta-analysis of randomized trials of vasoconstrictors assessing the association between changes in serum creatinine, taken as a continuous variable, and mortality, both while on treatment and during the follow-up period for survivors.

**Methods:**

The electronic databases of PubMed, Web of Science and Embase were searched for randomized trials evaluating the efficacy of vasoconstrictor therapy for treatment of HRS type 1 or 2. The relative risk (RR) for mortality was calculated against delta creatinine. The proportion of treatment effect explained (PTE) was calculated for delta creatinine.

**Results:**

Seven trials enrolling 345 patients were included. The correlation between delta creatinine and ln (RR) was moderately good (R^2^ = 0.61). The intercept and parameter estimate indicated a fall in creatinine while on treatment of 1 mg/dL resulted in a 27% reduction in RR for mortality compared to the control arm. In patients surviving the treatment period, a fall in creatinine while on treatment of 1 mg/dL resulted in a 16% reduction in RR for post-treatment mortality during follow-up. The PTE of delta creatinine for overall mortality was 0.91 and 0.26 for post-treatment mortality.

**Conclusions:**

Changes in serum creatinine in response to vasoconstrictor therapy appear to be a valid surrogate for mortality, even in the period following the completion of treatment.

## Introduction

Acute kidney injury (AKI) is a frequent complication of cirrhosis, occurring in up to 20% of hospitalizations [1], and associates with poor outcomes [2]. The most common etiologies for AKI in this setting are pre-renal azotemia, acute tubular necrosis and hepatorenal syndrome (HRS). Outcomes vary by AKI etiology, with the highest mortality seen in patients with HRS [3,4]. Despite a historically grim outlook, advances in treatment have provided hope for patients with HRS. The use of systemic vasoconstrictors, which act by ameliorating splanchnic and systemic vasodilation thereby attenuating renal vasoconstriction and restoring renal perfusion, has improved outcomes in what was once a near universally fatal disease [5]. The most common metric for evaluating efficacy of new therapeutics for HRS is “reversal of HRS” which has been traditionally and arbitrarily defined as achieving a fall in serum creatinine to below 1.5 mg/dL [6]. Multiple trials have confirmed that such a dramatic renal response to treatment among patients with HRS is indeed associated with reduced mortality [7–9]. Although vasoconstrictors are clearly useful in temporizing renal dysfunction, the ultimate definitive treatment for HRS remains liver transplantation [10,11]. As such, vasoconstrictor therapy should not be construed as an attempt at cure but in fact a bridge until such time as a patient is eligible and stable enough for transplant.

With the ultimate goal of treatment being bridging to transplant, even short-term improvements in renal function that are sufficient to stabilize a patient should, in theory, be beneficial. Patients who “respond” to therapy clearly have improved survival relative to non-responders. However, the arbitrary nature of the 1.5 mg/dL serum creatinine threshold for “response” limits the utility of this finding for determining the true association between lowering creatinine and mortality. For example, it has been shown that creatinine at the time of treatment initiation is a strong predictor of response [12]. This, however, is somewhat of a tautology; for example, it is not surprising that patients are more likely to have their creatinine fall under 1.5 mg/dL if they start treatment when it is at 2.5 mg/dL than they would be if treatment were initiated at 6 mg/dL. Demonstrating this, 33% of patients with creatinine between 2.5–3.0 mg/dL “respond” to placebo [12]. “Responders” then may include both those with the most dramatic falls in creatinine and those with the initially mildest degrees of renal dysfunction. However, the association between changes in serum creatinine and alterations in glomerular filtration rate is non-linear; a fall in serum creatinine from 2 mg/dL to 1.5 mg/dL is more meaningful than a fall from 4 mg/dL to 3.5 mg/dL. In light of the challenges of conducting studies in this critically ill population, the low prevalence of HRS and the difficulty of showing improvements in hard outcomes, the disease has been granted orphan disease status from the Food and Drug Administration (FDA) in order to expedite therapeutic development.

In order to have meaningful assessment of potential therapeutic interventions for HRS, trials may need to rely on continuous endpoints rather than the dichotomous distinction of “response” or “non-response”. Utilizing absolute or relative changes in creatinine in response to treatment as a surrogate outcome could better elucidate the association between lowering creatinine with treatment and mortality as well as simplifying the design and conduct of future trials. As defined by the Institute of Medicine (IOM), a valid surrogate is a biomarker that is intended to substitute for a clinical endpoint [13]. A surrogate endpoint is expected to predict clinical benefit (or harm or lack of benefit or harm) based on epidemiologic, therapeutic, pathophysiologic, or other scientific evidence. A critical and unanswered question then is to what extent generalized improvements in creatinine following treatment with vasoconstrictors are a good surrogate for improved survival.

We have performed a meta-analysis of randomized or quasi-randomized trials involving vasoconstrictors with or without albumin for the treatment of HRS. As our objective was to establish surrogacy, we have evaluated the association between changes in serum creatinine and both overall and post-treatment mortality.

## Methods

In order to ascertain the degree of association between change in creatinine and survival, we searched for treatment comparisons including (1) vasoconstrictor drugs alone or with albumin versus no intervention or albumin; (2) vasoconstrictor drugs alone or with albumin versus placebo or albumin alone or in combinations and (3) comparisons of different vasoconstrictor drugs. The decision was made to include studies with vasoconstrictor drugs in both the treatment and control arms because the goal of the study was not to identify the “optimal” treatment regimen. Rather, our aim was to see, when comparing two regimens, to what extent the difference in change in creatinine between arms associated with any difference in mortality. The electronic databases of PubMed, Web of Science and Embase were searched for publications between 1966 and June 2014 that evaluated the efficacy of vasoconstrictor therapy for the treatment of HRS type 1 or 2. We searched for articles with the key words “hepatorenal syndrome” or “HRS” and cross-referenced them with “vasoconstrictor therapy”, “dopamine”, “midodrine”, “octreotide”, “terlipressin”, “vasopressin”, “ornipressin”, “noradrenaline”, and “norepinephrine”, limiting the search to English and human subjects. The references of promising manuscripts were searched manually. Neither unpublished data nor abstracts were incorporated into the pool. The search generated a list of 615 unique publications. Criteria for eligibility for trial selection were: (1) involvement of human participants with a diagnosis of HRS, either type 1 or type 2, according to the definition by the International Ascites Club (as this definition has changed over time this refers to the accepted version at the time of the trial); (2) a prospective, randomized or quasi-randomized (pre-post) controlled trial evaluating the efficacy of a vasoconstrictor regimen for the treatment of HRS; (3) documentation of pre-treatment and post-treatment creatinine values; (4) documentation of mortality rates for treatment and control groups; (5) a period of follow-up after the completion of the treatment period. Exclusion criteria included trials with historical controls and cross-over trials. We excluded cross-over trials as interpreting the data from both periods of cross-over trials when evaluating a fluctuating biomarker can be challenging and each treatment period of identified trials was very brief.

**Fig 1:**
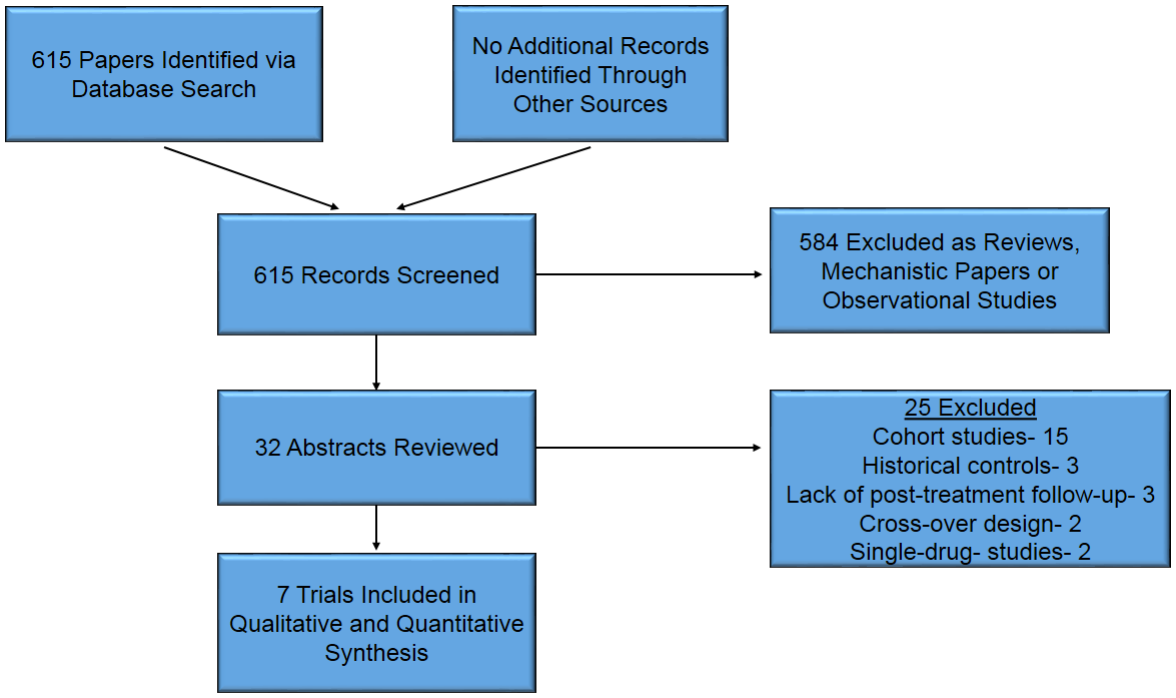
Identification of Trials for Inclusion in Review. Process and criteria by which papers were selected for inclusion in meta-analysis. Following exclusion of papers not relevant to the current study and those that met exclusion criteria, a total of 7 papers were included in the analysis.

583/615 (95%) manuscripts were excluded upon examining titles as consisting of reviews or relating to outcomes not pertinent to this analysis. 32 abstracts were reviewed by hand [14–45]. Of these, 15 were excluded as describing cohort studies [14–28], 3 employed historical controls [29–31], 2 utilized a cross-over design [32,33], 2 were excluded for comparing different regimens of a drug against itself [34,35] and 3 did not have any follow-up beyond completion of the trial period [36–38]. Seven trials were therefore selected for analysis [7–9,39–42]. The flow chart of the trial selection process is shown in Fig 1. Data was independently abstracted by two authors, J.B and C.P. Disagreement between reviewers was resolved in consultation with S.C. Data abstracted from the primary publications included treatment and control regimens, study size, type of HRS included, duration of treatment, serum creatinine and mean arterial pressure at the beginning and end of treatment, each trial’s definition of “response” to treatment, “response” rates for treatment and control arms, mortality rates for treatment and control arms during and after treatment and duration of follow-up. The potential for biases in each study was assessed as per the Cochrane Handbook for Systematic Reviews of Interventions [43]. The analysis and its reporting are done in accordance with PRISMA Checklist for meta-analyses (see S1 Checklist).

### Outcomes

**Fig 2:**
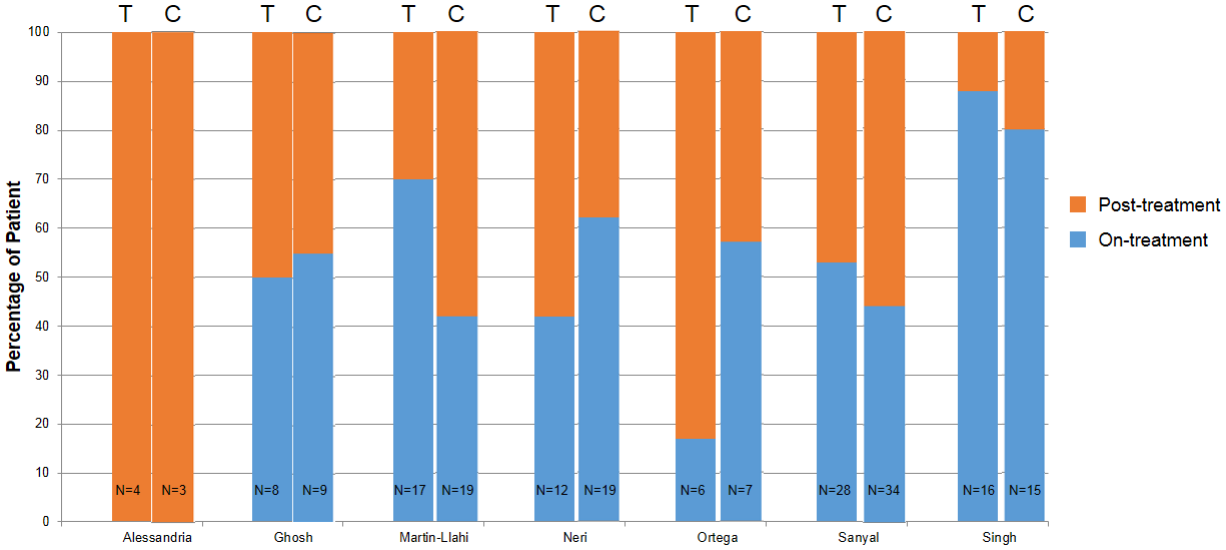
Proportion of Deaths Occurring During Intervention Period vs During Follow-up Period (Post-Treatment). Percentage of overall patients who died during the study period in the treatment arms (T) and the control arms (C) who died either while on treatment or during the post-treatment follow-up period.”N” indicates the total number of patients in each arm who died.

The primary outcome was post-treatment mortality and the primary analysis focused on the association between the degree of fall in creatinine while being treated for hepatorenal syndrome and post-treatment mortality. Because information on last serum creatinine was available only for patients who survived until the end of the trial period and was not available for those who died before completion of the trial period, we conducted the analysis in two ways. In the first analysis, we analyzed overall mortality from the start of treatment to the end of follow-up, including mortality during treatment as well as post-treatment mortality. In this scenario, the results would be biased towards the study hypothesis (demonstrating an association between change in serum creatinine and overall mortality) if there is differential mortality in the trial arms during the period of treatment. This is because creatinine at the time of death for such patients is likely to be higher than creatinine at the end of treatment for survivors and including these patients results in the inclusion of differential rates of deaths for patients who do not contribute to the determination of post-treatment creatinine. In the second analysis, we analyzed only post-treatment mortality (i.e., mortality from end of treatment to last follow-up in the study). This analysis therefore included only patients who survived the trial period and utilized serum creatinine from the last day of treatment. In this second analysis, the results will be biased away from the study hypothesis (towards the null) if patients with worsening creatinine died differentially at a higher rate during the (typically) two week treatment period because such patients, who would show a strong association between change in creatinine and mortality, are excluded. This phenomenon could not be captured as we do not have last creatinine before death in non-survivors in any of the published reports. Thus, the survivors in both arms are likely to be patients with comparatively lower creatinine, thereby reducing the separation in the final creatinine between the two arms. The proportion of overall and post-treatment mortality that occurred during the intervention period vs. during post-treatment follow-up for treatment and control patients for each study is shown in Fig 2.

### Statistical Analysis

The analyses were performed using RevMan version 5.3 (Nordic Cochrane Centre, Copenhagen, Denmark). Meta-analyses were performed using DerSimonian-Laird random-effects models due to expected heterogeneity. Results are presented as relative risks (RR) for binary and weighted mean differences for continuous outcomes, both with 95% confidence intervals (CI). I^2^ values were calculated as measures of the degree of inter-trial heterogeneity. Bubble plots were constructed and best-fit lines mapped for the natural log of the relative risk for death and the mean differences in delta creatinine. Pearson correlation coefficients were calculated for each plot. Forrest plots were constructed comparing treatment and control groups regarding delta creatinine from the beginning to end of the treatment period and mortality, both overall and post-treatment. For the sake of consistency, terlipressin combined with albumin was considered the treatment arm in each study as each included trial had at least one arm utilizing this regimen but, as stated previously, the intent is not to explicitly compare this regimen verses others. P values are 2-sided with a value <0.05 considered as statistically significant. RR are presented along with 95% confidence intervals.

### Calculation of Surrogate Effect

**Fig 3:**
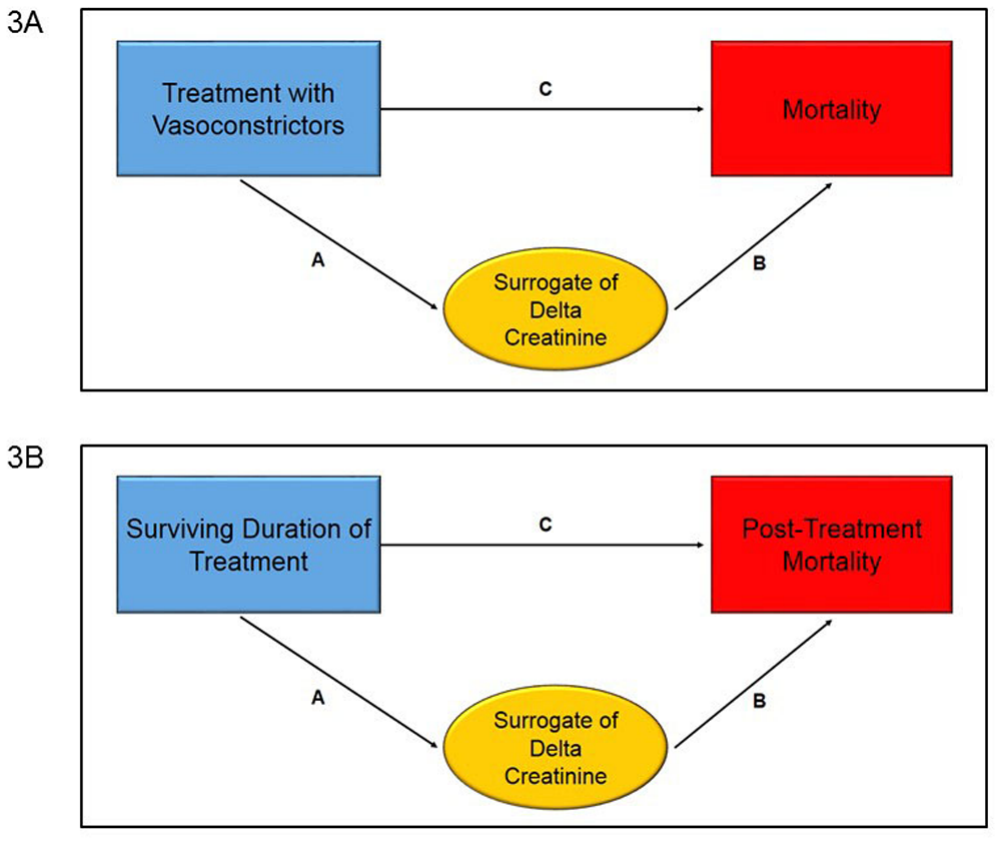
Path Graph for Proportion of Treatment Effect for Overall Mortality. Figure represents the path graph used to calculate the proportion of treatment effect (PTE) for overall mortality (3a) and post-treatment mortality (3b). The PTE, for both figures, calculates what proportion of the association “C” is attributable to the surrogate of delta creatinine while on treatment.

The purpose of this meta-analysis was not to evaluate the relative superiority of various vasoconstrictor regimens but instead to assess for the utility of utilizing change in creatinine as a surrogate for mortality. We sought to determine to what degree differences in changes in creatinine between randomized treatment groups, rather than the difference in treatment itself, explained differences in mortality. To calculate the surrogate effect of delta creatinine, we used the approach of proportion of treatment effect explained (PTE) (Fig 3a and 3b) and used the simple formula AB/C to calculate the surrogate effect, where A is the effect of the intervention on the delta creatinine, B is the effect of the delta creatinine on the mortality adjusting for intervention and C is the total effect of intervention on mortality [44–46].

## Results

### Trial Characteristic

**Fig 4:**
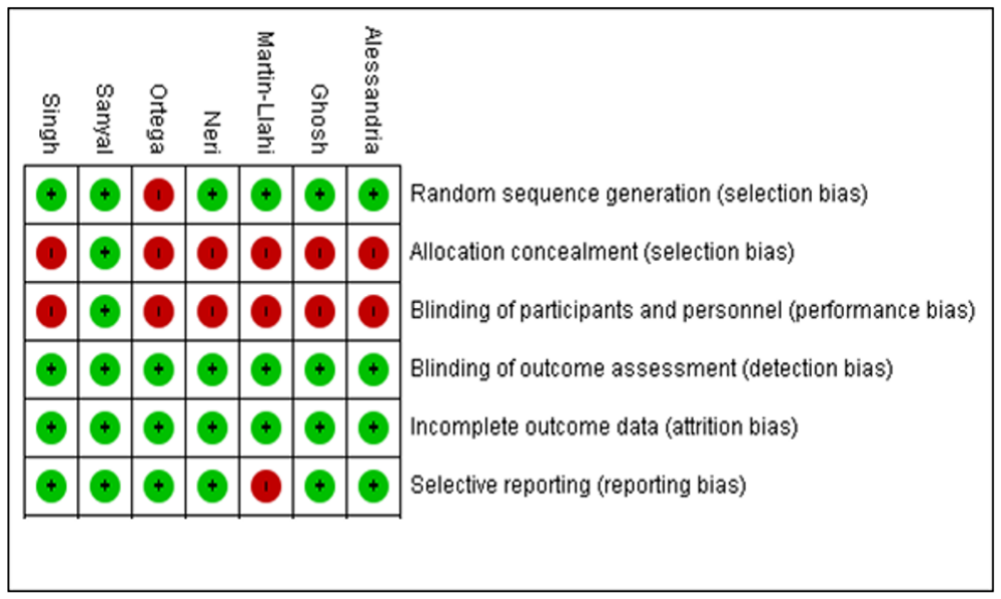
Assessment of Bias in Included Trials. Assessment for the risk of various biases in the included trials as recommended by the Cochrane Bias Methods Group. Green indicates low risk of bias, red indicates high risk of bias.

**Fig 5:**
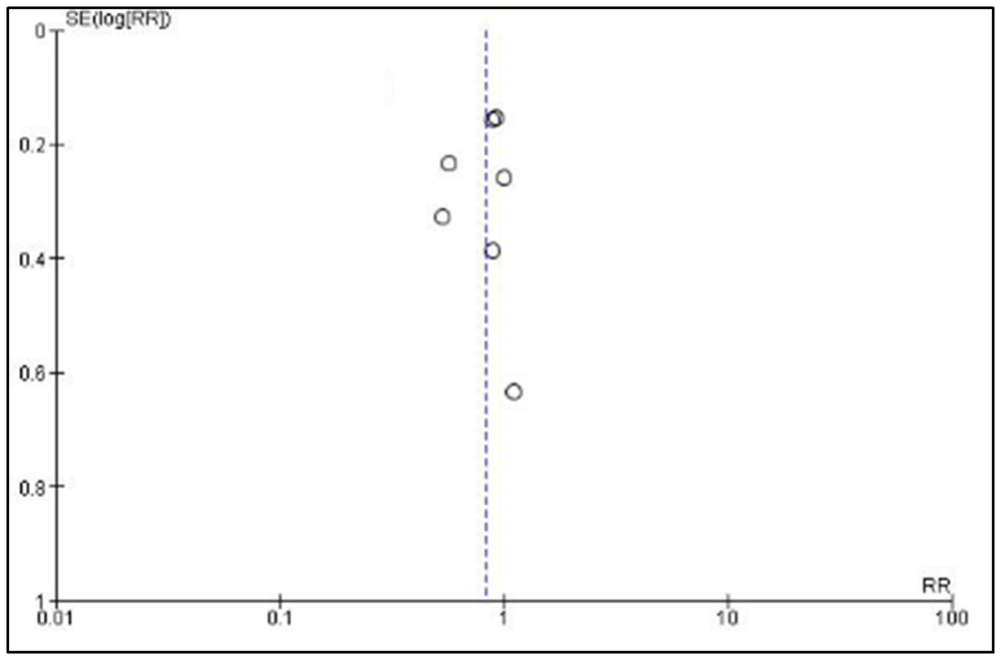
Assessment of Publication Bias. Assessment of potential publication bias among included trials via funnel plot.

The search of the literature identified 615 papers, of which 7 were eventually included in the analysis. Table 1 lists the general characteristics of the included trials. The series included publications between 2002 and 2013. Only 1 trial was conducted in the United States. Two were from India, two from Italy and two were from Spain, all were single centers. The total participants in the trials were 345, with 176 patients in what were considered the treatment arms and 169 in the controls. Terlipressin plus albumin was compared to noradrenaline plus albumin in 3 trials, albumin alone in 2, placebo plus albumin in one and compared to terlipressin alone in one trial. Initial doses of terlipressin ranged from 2mg daily to 6mg daily. 6/7 (86%) studies titrated terlipressin doses based on changes in serum creatinine and 1/7 (14%) had a pre-specified reduction in dose after 5 days of therapy [44]. The maximum allowable terlipressin dose ranged from 8mg to 12mg daily. The maximum duration of treatment was relatively consistent across the trials, ranging from 14 to 19 days. Three trials enrolled only patients with Type 1 HRS, one included only Type 2 HRS and the remaining three enrolled a mixture of Type 1 and Type 2. In general, the included trials had low risk for bias given the absence of poor randomization, incomplete data on outcomes or selective reporting. However, the majority of trials were non-blinded and without allocation concealment (Fig 4). Evaluation for publication bias via funnel plot (Fig 5) shows a potential paucity of smaller studies but, given the relatively small sample size of all published trials, it is possible this is artifactual [47].

**Table 1:** General Characteristic of Included Trials.

Author (Year)	Intervention	Control	HRS 1 vs 2	Definition of Response	Response %, Treatment vs Control
Alessandria (2007)	Terlipressin + albumin	Noradrenaline + albumin	9 HRS 1, 13 HRS 2	30% drop in Scr to < 1.5 mg/dL	83 vs 70
Ghosh (2013)	Terlipressin + albumin	Noradrenaline + albumin	HRS 2	Scr < 1.5 mg/dL	74 vs 74
Martín-Llahí (2008)	Terlipressin + albumin	Albumin	35 HRS 1, 11 HRS 2	Scr < 1.5 mg/dL	44 vs 9
Neri (2008)	Terlipressin + albumin	Albumin	HRS 1	Scr < 1.5 mg/dL	80 vs 19
Ortega (2002)	Terlipressin + albumin	Terlipressin	16 HRS 1, 5 HRS 2	Scr < 1.5 mg/dL	77 vs 25
Sanyal (2008)	Terlipressin + albumin	Placebo + albumin	HRS 1	Scr < 1.5 mg/dL	34 vs 13
Singh (2012)	Terlipressin + albumin	Noradrenaline + albumin	HRS 1	Scr < 1.5 mg/dL	39 vs 43

Abbreviations: HRS, hepatorenal syndrome; Scr, serum creatinine

**Fig 6:**
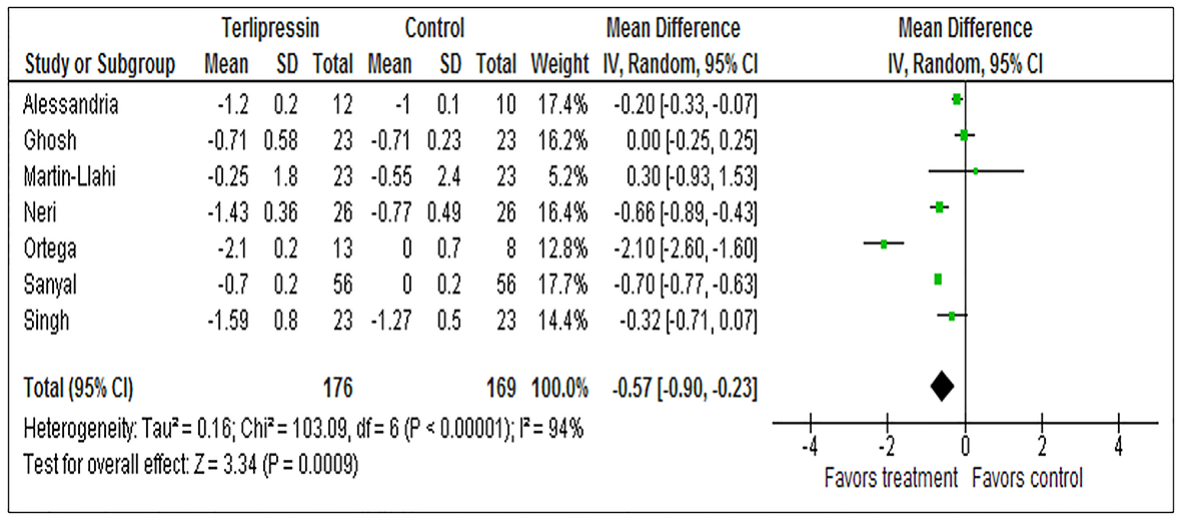
Change in Serum Creatinine in Survivors at the End of Treatment. Forrest plot assessing the weighted mean difference between delta creatinine in patients who survived treatment between those treated with terlipressin plus albumin vs all other control groups.

**Fig 7:**
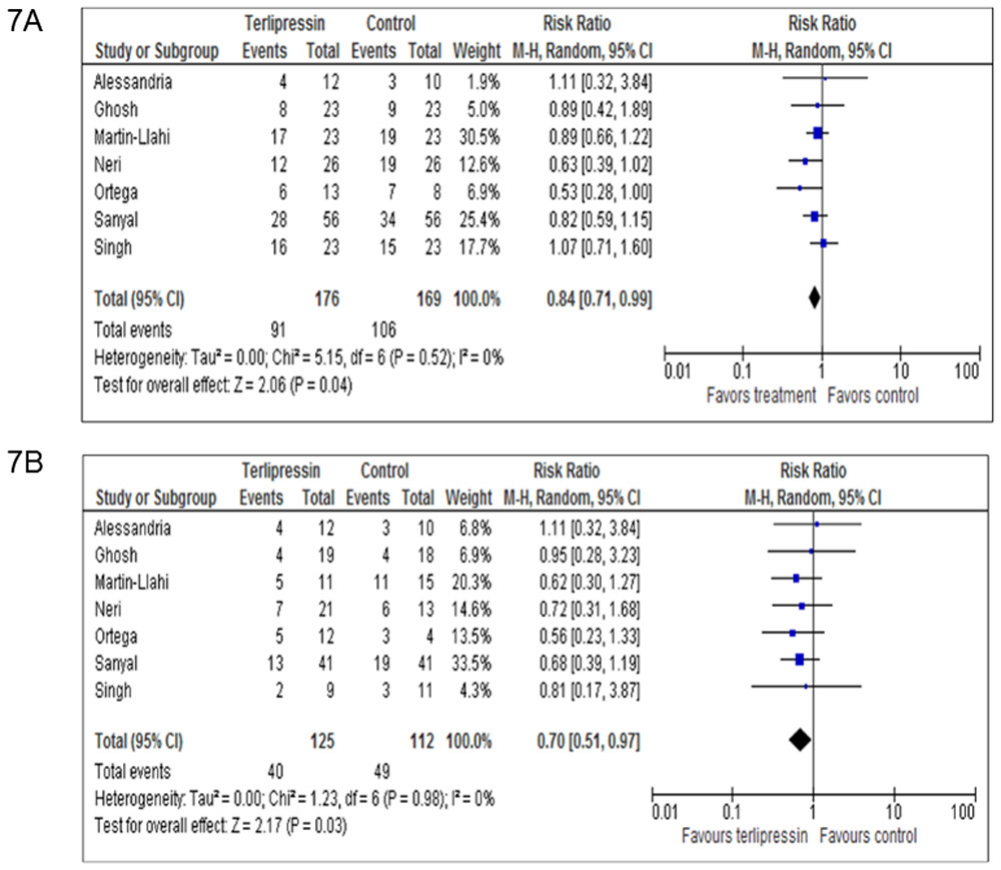
Mortality in Treatment vs Control Group. Forrest plots assessing the relative risk for overall (6a) and post-treatment (6b) mortality between those treated with terlipressin plus albumin vs all other control groups. Post-treatment mortality represents the mortality during the duration of the follow-up period in those who survived the treatment period. Total N therefore reflects those patients who survived the treatment period and “Events” are the number of those patients who then subsequently died during follow-up.

The starting and ending creatinine for the treatment and control arms of each trial as well corresponding overall and post-treatment mortality in each arm are shown in Table 2. The duration of follow-up for the studies was 3 months with the exception of Singh et al. (1 month). The primary analysis in the trial by Sanyal et al. included a 6 month follow-up but we utilized data from the manuscript to calculate 3 month mortality for consistency across studies. The serum creatinine level of patients in the treatment arms fell by an average of 1.14 mg/dL as compared to 0.61 mg/dL in the control arms among those who survived to the end of the treatment period for a mean difference of -0.57 (95% CI -0.90 to -0.23) (Fig 6). Importantly, overall mortality was also reduced in the treatment arm, RR 0.84 (95% CI 0.71–0.99) (Fig 7a). The combination of these findings suggests a fall in the RR for overall mortality of 16% for each reduction of creatinine of 0.57 mg/dL in patients treated with vasoconstrictors for HRS.

**Table 2:** Trial Outcomes.

	Scr at Baseline (mg/dL)				Scr at End of Treatment (mg/dL)				Overall Mortality		Post-Treatment Mortality		Duration of Follow-up
	N	Treatment	N	Control	N	Treatment	N	Control	Treatment	Control	Treatment	Control	
Alessandria	12	2.5 ± 0.3	10	2.3 ± 0.2	12	1.3 ± 0.2	10	1.3 ± 0.1	4/12	3/10	4/12	3/10	3 months
Ghosh	23	2.12 ± 0.21	23	1.98 ± 0.19	19	1.41 ± 0.58	18	1.27 ± 0.23	8/23	9/23	4/19	4/18	3 months
Martín-Llahí	23	3.60 ± 1.40	23	4.10 ± 2.40	11	3.33 ± 1.67	15	3.87 ± 2.40	17/23	19/23	5/11	11/15	3 months
Neri	26	2.80 ± 1.09	26	2.90 ± 1.19	21	1.27 ± 0.36	13	2.13 ± 0.49	12/26	19/26	7/21	6/13	3 months
Ortega	13	3.6 ± 0.5	8	3.4 ± 0.3	12	1.5 ± 0.2	4	3.4 ± 0.7	6/13	7/8	5/12	3/4	3 months
Sanyal	56	3.96 ± 2.19	56	3.85 ± 1.17	41	3.26 ± 2.19	41	3.85 ± 1.17	28/56	34/56	13/41	19/41	3 months
Singh	23	3.26 ± 0.81	23	2.82 ± 0.3	9	1.67 ± 0.92	11	1.55 ± 0.5	16/23	15/23	2/9	3/11	1 month

Abbreviations: Scr, serum creatinine; mg, milligram; dL, deciliter

Post-treatment mortality (mortality during follow-up for those who survived the intervention period) was also reduced in the treatment arm, RR 0.70 (95% CI 0.51–0.97) (Fig 7b). This finding suggests a greater fall in the RR for post-treatment mortality of 30% for each reduction of creatinine of 0.57 mg/dL in patients treated with vasoconstrictors for HRS who survive the treatment period.

**Fig 8:**
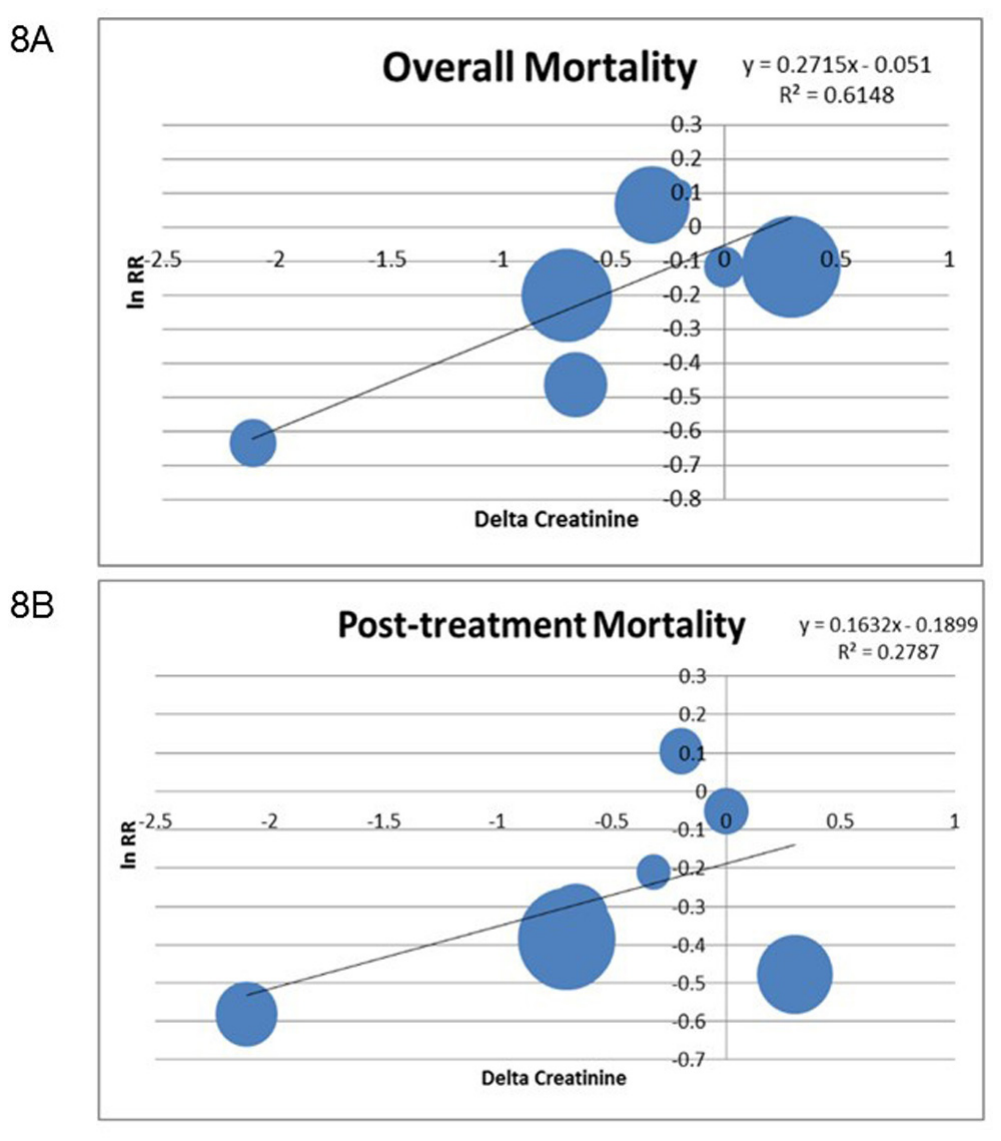
Correlation Between Change in Serum Creatinine During Treatment and Relative Risk of Overall Mortality. Bubbles graphs depict the correlation between changes in creatinine while on treatment with overall (7a) and post-treatment (7b) mortality. The intercept represents the fall in relative risk for mortality per each fall in serum creatinine of 1 mg/dL.

Utilizing meta-regression, the association between delta creatinine and the natural log of the RR for overall mortality was plotted (Fig 8a). The correlation between delta creatinine and ln (RR) was moderately good (R^2^ = 0.61). The resulting intercept and parameter estimate indicate that a fall in creatinine while on treatment of 1 mg/dL will result in a 27% reduction in RR for mortality compared to the control arm. However the correlation between the percent change in creatinine from baseline and ln (RR) was poorer, R^2^ = 0.30.

The association between delta creatinine and the natural log of the RR for post-treatment mortality was then plotted (Fig 8b). The correlation between delta creatinine and ln (RR) was significantly lower than for overall mortality, (R^2^ = 0.28). The intercept and parameter estimate indicate that a fall in creatinine while on treatment of 1 mg/dL will result in a 16% reduction in RR for post-treatment mortality compared to the control arm in those who survive.

### Assessment of Proportion of Treatment Effect Explained (PTE)

The difference of delta creatinine between intervention and control is -0.57 (A). Importantly, overall mortality was also reduced in the treatment arm with RR 0.84 and ln (RR) = -0.17 (C). The effect of delta creatinine on ln (RR) of mortality is 0.27 (B), then PTE for delta creatinine is 0.91 (calculation: 0.27 x (-0.57)/(-0.17)). The PTE for delta creatinine on post-treatment mortality was 0.26 (calculation: 0.16 x (-0.57)/(-0.35).

## Discussion

HRS has traditionally been one of the most feared complications of cirrhosis and, prior to the introduction of modern treatments, was associated with near universal mortality if patients did not rapidly receive a liver transplant. Over the past 20 years the treatment of HRS has improved with the introduction of systemic vasoconstrictors, which improve renal function by shunting blood from the splanchnic to systemic circulation. Critically however, approval of such agents, in particular terlipressin, has lagged in North America. Trials evaluating these agents have struggled to distinguish between the impacts of different treatment arms verses different treatment responses on survival. We have performed a meta-analysis of randomized trials of vasoconstrictors attempting to determine the attributable changes in mortality per unit change in creatinine. Statistically such an approach treats changes in creatinine itself as the intervention, irrespective of treatment regimen. To confirm the utility of delta creatinine as a surrogate outcome, we assessed the association between fall in creatinine and mortality both overall and post-treatment, thereby mitigating survivor bias.

The fall in creatinine was significantly larger in patients treated with terlipressin plus albumin, 1.14 mg/dL vs 0.061 mg/dL, than other regimens. Mortality was also reduced in these patients, RR 0.84 (0.71–0.99). Utilizing meta-regression, we determined that the correlation between delta creatinine and overall mortality was moderately good, R^2^ = 0.61 and that a fall in creatinine of 1 mg/dL while on treatment results in a 27% reduction in mortality. Remarkably, the PTE for the effect of delta creatinine on mortality was extremely strong, 0.91. Critically, though the associations were milder, delta creatinine while on treatment was also associated with mortality in survivors in the period after treatment was completed. A 1 mg/dL fall in creatinine while on treatment was associated with a 16% reduction in RR for mortality during the follow-up period and the PTE for this interaction was 0.26.

In line with the FDA’s directives, assessing changes in creatinine in response to HRS therapy as a continuous surrogate outcome may expedite development and approval of new therapeutics as these continuous endpoints would provide more statistical power than the dichotomous clinical end-points of mortality or reversal of HRS. To be valid and acceptable for drug development however a surrogate outcome must be directly linked to a hard outcome such as mortality. In AKI research, such a relationship is not always readily apparent and few studies have demonstrated falls in mortality with treatments designed to lower creatinine. For example, in the setting of cardiac surgery, performing surgery off pump as opposed to on pump lowered AKI rates but this did not correlate with improved survival [48]. However, in this study, while the incidence of AKI varied significantly between groups, the actual mean difference in serum creatinine between the treatment arms was much lower than what is seen in HRS trials.

Numerous observational cohort studies and, more recently, several randomized trials have shown improvements in renal function when patients with HRS are treated with vasoconstrictors [7,37,39]. Such trials however have primarily focused on comparing treatment regimens in their ability to lower creatinine to an arbitrarily established cutoff, typically 1.5 mg/dL. In regards to mortality, the focus of these trials has again been in comparing outcomes for different treatment regimens rather than specifically assessing the association between falls in creatinine and survival. In multiple meta-analyses, evidence for improved mortality with vasoconstrictor treatment has been elusive [49,50], though a recent Cochran review of randomized terlipressin trials does suggest reduced mortality in patients treated with terlipressin and albumin [51]. In contrast to these muddied findings, virtually all trials have found markedly improved survival in “responders” vs. “non-responders”, again typically defined by a fall in creatinine to below 1.5 mg/dL during the course of treatment. In the trial by Sanyal et al. a statistically significant improvement in survival for “responders” vs. “non-responders” was seen by 14 days, 100% vs 65%, p = 0.002, and persisted to at least 90 days (66% vs 36%, p = 0.025) [38].

However, hospitalized patients with decompensated cirrhosis have a very high mortality. Death from non-renal causes is a competing risk for renal recovery; responders by definition have to live long enough to respond. Assessing the association between response and mortality therefore is inherently biased in favor of showing treatment benefit, evidenced by the extremely high PTE of 0.91 seen in our analysis for the interaction between delta creatinine and overall mortality. It is not sufficient then to note that patients who have a dramatic fall in serum creatinine when treated with vasoconstrictors have reduced mortality. Our finding that *post-treatment* mortality among those who survive the study period is also associated with delta creatinine while on treatment is therefore both novel and critical and suggests delta creatinine may indeed be a valid surrogate for short-medium term mortality among patients with HRS treated with vasoconstrictors.

Our study is not without limitations. Despite a rigorous search of the literature, the number of identified trails meeting the inclusion criteria was low and the resulting number of included patients was modest. This number was further reduced when evaluating post-treatment mortality due to the high mortality rate during the intervention period. Ideally, such patients could have been included utilizing time varying analysis but creatinine values at the time of death for those patients who died prior to the completion of the intervention period were unfortunately not available, nor was information available as to on which study day these patients died. In two trials exact numbers regarding post-treatment mortality were not provided in the text but had to be extrapolated from figures. The included studies show large heterogeneity when assessing for changes in creatinine at the end of treatment but very little heterogeneity in regards to the association between changes in serum creatinine and both overall and post-treatment mortality. Meta-regressions usually, though not always [52,53] involve at least 10 studies. The findings from this aspect of our analysis therefore must be taken as preliminary pending the availability of more trials. Finally, studies varied in their inclusion of patients with Type 1 vs. Type 2 HRS and such patients may be expected to differ in respect to their associations between changes in creatinine and mortality.

In conclusion, improvement in serum creatinine while receiving vasoconstrictor therapy for HRS is associated with both overall and post-treatment mortality. Rather than approaching response to treatment with a dichotomous, arbitrary creatinine cutoff, these results suggest that treating the degree of response as a continuous variable may add more nuance and provide a valid surrogate for mortality in this extremely challenging to study patient population. Such a surrogate could improve the assessment of utility of current treatments and facilitate expedited approval of novel therapies.

## Supporting Information

S1 Checklist Checklist for analysis and reporting of meta-analyses as per the Cochrane Handbook for Systematic Reviews of interventions. (DOC)
